# Health Promotion Values Underlying Healthy Eating Strategies in The Netherlands

**DOI:** 10.3390/ijerph20146406

**Published:** 2023-07-20

**Authors:** Christina Gillies, Hedwig te Molder, Annemarie Wagemakers

**Affiliations:** 1 Strategic Communication Chair Group, Department of Social Sciences, Wageningen University & Research, 6706 KN Wageningen, The Netherlands; 2 Provincial Population & Public Health, Alberta Health Services, Edmonton, AB T5J 3E4, Canada; 3 Department of Language, Literature, and Communication, Faculty of Humanities, Vrije Universiteit Amsterdam, 1081 HV Amsterdam, The Netherlands; h.f.m.te.molder@vu.nl; 4 Health and Society Chair Group, Department of Social Sciences, Wageningen University & Research, 6706 KN Wageningen, The Netherlands; annemarie.wagemakers@wur.nl

**Keywords:** values, nutrition, health promotion, health professionals, The Netherlands, qualitative research

## Abstract

Healthy eating strategies are a large focus of research, practice, and policy in the Netherlands to improve the diets of socioeconomically disadvantaged populations (SDPs) and reduce health inequalities. However, the fundamental values of the health professionals that develop, implement, and evaluate healthy eating strategies are not explicit. Understanding and challenging these values may be an important step in aligning and improving efforts to support healthy diets in SDPs. The purpose of this qualitative study was to critically examine the values influencing strategies to promote healthy eating in SDPs in the Netherlands. In-depth interviews guided by a critical health promotion model were conducted with a diverse group of health professionals (*n* = 29) between October 2020 and January 2021 and analyzed using reflective thematic analysis. Results indicated that health professionals’ values overlapped in many ways, including their shared values concerning beneficence, responsibility, and collaboration. However, value conflicts were also uncovered surrounding assumptions about SDPs and ethical change processes. The co-existence of conventional and holistic health promotion values also reflected an enduring emphasis on individual-level healthy eating strategies. It is concluded that ongoing attention to the values of health professionals is needed to advance healthy eating strategies and reduce diet-related health inequalities.

## 1. Introduction

Diet-related health inequalities among socioeconomically disadvantaged populations (SDPs)—people living in less favourable social and economic circumstances relative to others in the same population at a given time—are well-reported in many European countries. In the Netherlands, SDPs that have been prioritized in health promotion include low-income and/or low-educated Dutch-born individuals as well as Turkish and Moroccan migrants [1]. Due in large part to factors and conditions within their daily social, economic, and physical environments, SDPs are more likely to consume lower-quality diets and have poorer nutrition-related health outcomes [2,3,4]. SDPs also tend to have higher incidence, morbidity, and mortality rates for diet-related chronic diseases, including cardiovascular disease, type II diabetes, and certain cancers [5,6]. As such, it is important that effective strategies to support healthy eating be developed to improve the dietary behaviours of populations and ultimately reduce nutrition-related health inequalities.

Healthy eating strategies are a large focus of research, practice, and policy at the global level [7] as well as in many countries, including the United States [8], Canada [9], and the Netherlands [10,11]. A healthy eating strategy can be defined as an organized effort intended to result in significant and sustainable changes in the dietary behaviours of an identified group and/or entire population in the pursuit of better health and healthy weights [12,13]. Healthy eating strategies may focus on individual factors (e.g., food skills, knowledge, and beliefs) and/or address the broader social, physical, and policy-level determinants (e.g., food affordability, availability, and quality) [12]. A number of lifestyle (and combined lifestyle) strategies have targeted SDPs in the Netherlands by supporting healthy eating through education and awareness courses and improvements to the food and beverages available in school environments [14,15]. These strategies are also often part of a broader strategy to promote healthy lifestyles and reduce health inequalities [11].

In pursuit of these goals, health promotion professionals must attend to complex issues and multiple determinants of eating behaviors, which requires the ability to understand and work explicitly with the values and principles of modern practice [16,17]. In this context, ‘values’ are defined as ideas or concepts that are regarded as worthy, desirable, or useful to health promotion, while ‘principles’ describe the actions that are required to enact or attain the values [17]. Daily decisions concerning who to focus on, what to implement, and how to evaluate the success of healthy eating strategies are underpinned by individual and collective values with moral and ethical implications [18]. Social values surrounding social justice and equity, for example, influence the degree to which health promotion professionals prioritize action with communities that are most socioeconomically disadvantaged when developing healthy eating strategies.

While a wide body of the literature has focused on identifying the factors influencing eating behaviours in SDPs [19,20,21,22], much less attention has been paid to exploring the values that underpin health promotion practice. In recent decades, there has been increased recognition of the need for professionals involved in developing, implementing, and evaluating public health strategies to critically reflect on the values and principles inherent in these strategies to promote professional accountability and drive change towards best practice [18,23,24]. However, there remains a lack of empirical research concerning the values of health professionals and the way that health promotion values are enacted in their everyday assumptions, practices, and decisions. The aim of this qualitative study is to critically examine the health promotion values influencing strategies to promote healthy eating in socioeconomically disadvantaged populations (SDPs) in the Netherlands.

## 2. Materials and Methods

This study is the larger of two studies aiming to explore, identify, and operationalize the values of nutrition health professionals in the Netherlands. In a sub-study, our team developed the Values Wheel of Nutrition Health Professionals (VWNHP) [25] by organizing health professionals’ key values according to a set of basic human values and operationalizing them for critical reflection. In this study, an explorative qualitative design was used to elicit the values underlying healthy eating strategies for SDPs in the Netherlands within the context of critical health promotion.

The Red Lotus Critical Health Promotion Model (RLCHPM) [26,27] was used as a conceptual framework for the study. First published as the Red Lotus Health Promotion Model [28], the RLCHPM explicitly incorporates a system of current health promotion values and principles and is designed to support critical health promotion practice. Represented as the stem of the Red Lotus plant, the values and principles of the RLCHPM are organized into three domains (i.e., philosophical, ethical, and technical) that collectively make up a dynamic system. These may be contrasted with the values and principles of conventional health promotion [17]. The model incorporates a heuristic process that supports health professionals in identifying their position on the continuum between current and conventional health promotion [27] and assessing the degree of congruency between best and current practice.

A purposive sample of key informants was selected using a maximum variation sampling to capture a wide range of perspectives [29]. As health promotion evolved out of clinical and settings-based work in health education and disease prevention [30], we sought the perspectives of a diverse group of professionals, including general practitioners, dietitians, and individuals from academic institutions, health promoting institutions, and municipal health services. We recognize the considerable diversity within and between these groups and acknowledge that many of these individuals may not self-identify as a ‘health professional’. However, all are referred to as such (or simply ‘professionals’) in this paper to allow for clearer reporting of findings among the collective group.

Health professionals were recruited through the personal network of the researchers. As several professionals declined due to lack of time (*n* = 8) or did not respond (*n* = 8), web searches of relevant organizations were used to identify potential participants. Professionals were invited to participate via email, with telephone follow-up if necessary. Recruitment continued until theoretical saturation had been reached; more specifically, this was the point at which themes were well-developed, and no new or discrepant information was found in the data [29].

In-depth, semi-structured interviews were conducted from October 2020 to January 2021 by an experienced qualitative researcher (native English speaker) accompanied by a research assistant (native Dutch speaker), in case the occasional translation was necessary, or solely by a trained research assistant (native Dutch speaker). A semi-structured interview guide was developed using the principles of Appreciative Inquiry (AI)—a strength-based approach that makes use of storytelling to explore participants’ underlying values [31,32]—and values found in the RLCHPM. The interview guide was pilot tested with two health professionals to ensure the questions were comprehensible and effective at eliciting values. As only one minor wording change was made to the interview guide, these two interviews were included in the data analysis. All participants resided in the Netherlands and were interviewed using online audio-visual communication software. Interviews were conducted in English or Dutch—according to the participant’s preference—and recorded after providing verbal consent. The interviewers also recorded field notes at the end of each interview to capture contextual information and allow for reflection on their own personal biases and assumptions. On average, interviews lasted 41 min (ranging from 33:00 to 1:10).

Interview recordings were transcribed verbatim and verified by the original interviewers. Dutch transcripts were translated into English and verified a second time. Anonymized interview transcripts were loaded in Atlas.ti qualitative analysis software, and reflexive thematic analysis (TA) was used to explore the values of health professionals. Following the approach developed by Braun and Clarke [33], the analysis was completed in a six-phase recursive process: (1) familiarization: one researcher read interview transcripts several times; (2) generating codes: with an inductive orientation, the same researcher coded the entire dataset using semantic and latent coding; (3) generating initial themes: codes were examined to identify significant and recurring patterns of meaning; (4) reviewing themes: themes were discussed among all researchers and checked against the dataset to ensure they were well-supported; (5) defining and naming themes: after consensus was reached, a detailed analysis of each theme was further developed by one researcher; and (6) writing up: narrative and data excerpts were weaved together, highlighting the similarities and differences between professionals’ values.

In reflexive TA, researchers have a central role in the knowledge production process and are engaged in interpreting the data through their own social lens, theoretical assumptions, and scholarly knowledge [33]. As such, it is important to recognize that the first author who analyzed the data in Phases 1–6 is an anthropologist with strong social justice values and a commitment to participatory research approaches. The researchers who took part in Phase 4 include a researcher who specializes in uncovering values in people’s real-life talk (discourse) about health and a researcher in the field of community health promotion with a focus on the inclusion of stakeholders in research and practice. Prior to submission, a draft of the manuscript was shared with all interviewed professionals, who were given the opportunity to comment on the authors’ interpretations of the findings. No changes were requested.

## 3. Results

In the following sections, health promotion values are situated within the three domains of the RLCHPM: (1) philosophical values, which inform what is important and what should be enacted to improve healthy eating in SDPs; (2) ethical values, which provide guidance on the humane or ethical way to change people’s eating behaviors; and (3) technical values, which pertain to actions required to promote healthy eating. Illustrative quotations include profession and participant code—‘D’ denotes an interview conducted in Dutch, while ‘E’ denotes an interview conducted in English. An overview of the characteristics of participants (*n* = 29) can be found in Table 1. No differences in the values and beliefs expressed by participants based on profession were found during the data analysis.

### 3.1. Philosophical Values

#### 3.1.1. Framing the Issue

Health professionals explained that the issue of (un)healthy eating is complex and influenced by many individual and environmental factors. As one professional explained, “Health behaviors are so much formed by your upbringing, your social environment, your physical environment, your economic possibility” [E05, A]. Most explained that healthy eating was unaffordable for SDPs and that the price of nutrient-dense foods like fruit and vegetables was too high in relation to calorie-dense, nutrient-poor convenience foods. However, not all professionals agreed that nutrient-dense foods were more expensive and instead framed the issue in terms of knowledge on how to budget and source more affordable foods.

Many health professionals also believed that issues such as poverty, stress, and addiction had a much larger influence on eating behaviors than individual choices. Accordingly, most professionals believed that the issue of unhealthy eating in SDPs should not be framed in terms of eating behaviors at all. Rather, they suggested that it was important to help people overcome barriers to health in general by tackling issues like low income, isolation, and unsafe living conditions. As one professional stated, “We have to solve the personal problems first that come before a healthy lifestyle. So financial crises, poverty, debts […] all those kinds of things we need to tackle first before we can talk about ‘okay it is important for you to eat healthy’” [E03, A/HP].

#### 3.1.2. Assumptions

Most health professionals believed that SDPs were motivated to eat healthy foods but that their lives were complicated by other priorities or priorities that differed from those of health professionals. As one professional said: “I think there’s nobody in this world who doesn’t want to be healthy […] It’s just very often there is no room for people to be healthy and to engage in those behaviors” [E01, A]. Some professionals also commented that it is often assumed that SDPs have the same backgrounds, behaviors, and issues. Accordingly, they noted that it was problematic to stigmatize SDPs: “I don’t think low SES equals poor nutrition […] we have to be a bit careful with that” [D15, GP].

Although most professionals assumed that SDPs naturally desire to do the best they can, others stated that SDPs lacked motivation, made poor financial decisions, and needed guidance to improve their behaviors. These professionals were critical or skeptical of the choices made by SDPs, even when they recognized that they lived complicated lives. As one professional remarked: “Smoking is always wasted money of course. Do they have pets? Then on the one hand you have no money for your food, but you have pets […] those are the people who tattoo a lot, that I think ‘okay you don’t have that kind of money but still you do it and your own body, your own liver and heart, are completely fat, what are you doing?” [D07, D].

#### 3.1.3. Approach to Health

By recognizing the complexities surrounding the eating behaviors of SDPs, some professionals focused on developing strengths-based, empowering strategies that enabled people to gain better control over their lives. As one explained, “I find it very nice to work in that way because you, you immediately start looking at where is someone’s strength. You don’t actually look at where the problem is, you just look at where the solution is” [D15, GP]. However, these approaches were largely aspirational and lacking in real-world practice. As one health professional remarked, “Although [professionals] confess that they want it differently, they think about health promotion along very traditional lines of thought” [D08, MHS].

Far fewer professionals valued strategies that sought to educate people about their ‘unhealthy’ behaviors and focused on the negative aspects of individuals or communities. Nevertheless, deficit- and fear-based approaches were valued by a few professionals who believed they were an effective way to convince SDPs to commit to improving their eating behaviors. As one explained, “Talking about diabetes triggers something, because they have an aunt, or a grandma, or a sister, or a brother who has diabetes and they don’t want their children to end up like them” [E08, MHS].

### 3.2. Ethical Values

#### 3.2.1. Beneficence

Health professionals prioritized work with people experiencing the most socioeconomic disadvantage relative to others in the same population. They valued maximizing beneficence and believed that SDPs were less likely to benefit from whole-of-population strategies such as nutrition education campaigns. As one remarked, “When you do address the population as a whole, then you don’t get the people that really need it” [E06, A]. Accordingly, some professionals were quite critical of seeking benefits among all groups in society and the negative outcomes this approach could have on SDPs. As one professional explained, “I know that in Amsterdam there have been whole strategies to improve the nutrition […] and ultimately you find that the gains are greatest in the group with higher SES and living in a better neighborhood than in the neighborhood where ultimately not so much changes [D15, GP]”.

#### 3.2.2. Power and Responsibility

Health professionals shared a belief that people from SDPs experience considerable barriers to healthy eating that limits their ability to improve their eating behaviors. As one stated, “Putting the responsibility in their hands is too much because being already from a low socioeconomic position means you have so many struggles that we not in that position cannot think about, so food and nutrition strategies are the last thing on your mind” [E07, A]. Rather, health professionals believed that the responsibility to improve the diet and nutrient status of SDPs ultimately fell on government officials. In addition, professionals were united in their commitment to addressing health issues in SDPs through their different roles. Echoing the sentiments of many others, one professional explained, “I like to help people […] And when I was thinking of what I wanted to become when I grew older, I want to do something concerning health to help people to yeah, live a better life” [E13, MHS]. Despite these intentions, however, some professionals—particularly GPs—doubted whether they held the power to influence change: “I am also a bit discouraged, but I think a lot of care providers are like, ‘What are you going to do about it’? You do run your riot but still a certain hopelessness of ‘am I still going to make a difference?’” [D16, GP].

#### 3.2.3. Change Processes

Although they were unified in many ethical values, health professionals differed in their values concerning ethical ways to create change. Some professionals valued ‘top down’, expert-driven change processes that positioned people in SDPs as passive recipients of external decisions. For instance, some believed in ‘nudging’ people by making subtle environmental changes to shape behavior. Others suggested more overt practices by ‘forcing’ people to make the ‘right’ choice: “Don’t leave everything to as a free choice, because a lot of people don’t have that free choice or don’t know which one to choose” [E12, MHS]. In contrast, others valued ‘bottom-up’ participatory change processes that sought the perspectives of people from SDPs. Rather than being coercive, these change processes were valued for being respectful of personal autonomy. However, participatory approaches appeared to be largely aspirational as current examples of these change processes in professionals’ work reflected low levels of participation from SDPs—such as ‘involving’, ‘working with’, and ‘talking to’ people. As one professional remarked, “Sometimes we have an expert meeting and then we ask opinions and then we still design the intervention from behind our desk and there are really examples in which the community themselves design the intervention” [E01, A].

### 3.3. Technical Values

#### 3.3.1. Strategies to Improve Healthy Eating

Most professionals implemented individual-level strategies such as nutrition education to improve healthy eating in SDPs by addressing immediate behavioral risk factors and improving people’s knowledge and skills. However, most professionals concurrently emphasized the value of strategies that attend to multiple determinants in the social and structural environments of SDPs. As one described, “Look, it eventually starts with education. But of course […] that that alone is not enough” [D02, MHS]. For instance, all professionals supported strategies that attended to structural determinants by taxing certain foods and subsidizing others. As one remarked, “Well, there has been some talk of a sugar tax, so that there is less sugar in soft drinks and also to make fruit and vegetables cheaper, that would be very nice” [D09, GP].

#### 3.3.2. Collaboration

Health professionals were unified in their belief that improving healthy eating among SDPs would require collaboration between different actors and organizations. Accordingly, all professionals valued interdisciplinary learning, diversity, and teamwork. Reflecting on the value of collaboration, one professional stated: “I think it broadens your perspective on things, because when you work just within your own team you kind of see things from your own view […] but I think that if you really work together with people, it sometimes brings some challenges or insights that can really help you do your work better” [E13, MHS].

Several professionals remarked that the shared value of collaboration was a strength in the Netherlands and was one that could be leveraged to improve eating behaviours. Nevertheless, it was recognized that healthy eating strategies are still fragmented, and there needs to be greater emphasis on collaboration and integration of different perspectives in practice. As one professional explained, “We need to do it together. And for some reason we’re still not able to do that […] joining our forces in terms of our disciplines and in terms of our knowledge is something that we should do. I think we might be amazed about the amount of knowledge that already exists, but we’re just not able to combine in the right way” [E08, MHS].

## 4. Discussion

To our knowledge, this is the first study to apply a conceptual framework of critical health promotion to study the values underlying healthy eating strategies. In doing so, this study has helped to reveal how values drive daily practice and decision-making surrounding strategies to improve the eating behaviours of SDPs in the Netherlands. First, our findings indicate that there may be a need to address the stigmatization of SDPs among health professionals. Despite consistently framing the issue of healthy eating as complex and influenced by factors outside of individual control, some health professionals were nevertheless critical of the choices made by SDPs. This finding indicates that health professionals may believe there is a ‘right’ way for individuals to allocate their resources and that failure to do so indicates a lack of motivation or capability. Research has shown that SDPs perceive the stigma of being responsible for their own situations and anticipate negative social reactions and judgements from others [34,35]. A recent study found that people living in a disadvantaged neighborhood in the Netherlands perceived the discrepancy between public health messages that suggest that healthy food consumption is a free choice and their limited ability to conform to these messages [36]. Strategies to improve healthy eating in SDPs could thus unintentionally result in stigmatization by assuming that certain practices characterize all members of a group and attributing responsibility to those who may have little control over their environmental conditions [37].

Related to this dualism, our findings suggest that there is a continued emphasis on ‘downstream’ strategies aimed at changing individual health behaviors in the Netherlands. International evidence suggests that ‘upstream’ strategies that address social and structural factors and require less individual agency and resources are needed to promote healthy eating and improve the conditions that influence nutrition inequalities [5,38]. Most health professionals in this study implemented individual-level strategies despite recognizing the importance of action at different environmental levels. As such, the shared value of social responsibility for health identified in this study appears to not yet be reflected in current healthy eating strategies adopted in the practice of participants. Indeed, neoliberal and holistic conceptualizations of health co-exist in public health discourses in the Netherlands and translate into an emphasis on individuals being responsible for their own health despite simultaneous recognition that health is also a product of social conditions [11,36]. This finding also stands in contrast to professionals’ shared value of beneficence, as individual-level strategies are known to be less effective in SDPs [39]. However, it is possible that the values expressed in the technical domain may be reflective of a lack of interest, understanding, or knowledge of the socioecological science, the scope of the profession, or the lack of capacity and resources to pursue more comprehensive healthy eating strategies among the participants.

Despite being committed to making a difference in the lives of SDPs, some professionals also doubted their ability to create change. Health professionals are undeniably limited in the actions they can take to improve healthy eating due to factors like time, resources, and professional boundaries that may leave them feeling disempowered. However, there are ways for health professionals to overcome barriers and contribute to changing the trajectory of current health promotion strategies to support healthy dietary behaviours. For instance, the shared value of collaboration identified in this study is a considerable strength that can be leveraged to clarify and align the strengths, priorities, and goals of different stakeholders. This research also highlights the potential for health professionals to advocate for policy changes to improve the prices and availability of healthy food. As intersectoral collaboration is required to make healthy foods more attractive, affordable, and accessible in relation to less healthy foods [40], realizing this shared value in practice is integral to planning new or improving existing healthy eating strategies.

To build on the value of collaboration identified in this study, we suggest that ongoing critical reflection on health promotion values is required. Critical reflection on the values that inform strategies to improve healthy eating in SDPs may offer health promotion practitioners, researchers, and policymakers the opportunity to understand how their underlying values drive daily practice and decision-making. This knowledge can set a common ground for reorienting collective healthy eating strategies for SDPs and taking joint ethical action on diet-related health inequalities. Without such reflection and awareness, ethical disconnects will occur, and action may not be consistent with best practice health promotion [16,24]. For instance, identifying participatory change processes as a value but ignoring them when developing new healthy eating strategies using a traditional ‘bottom up’ approach. It is important, however, not to fall into the fallacy of “blaming” individual health professionals and instructing them to do better, knowing that an integrative approach is essential for effect.

While it does not explicitly provide a tool for engaging in critical reflection, the RLCHPM that was used to guide this study has been demonstrated to be a useful reflection tool to guide health promotion practice [26]. This model can enable professionals to purposefully put into action a connected system of values across the phases of a health promotion process, including the development, implementation, and evaluation of healthy eating strategies [26]. Our team also developed the VWNHP [25], which can be used to further guide critical reflection by providing a common vocabulary for professionals in different institutions and organizations working to think and talk about their values. While other models have been proposed as appropriate for this purpose [41], empirical research is needed to understand how critical reflection can be operationalized to explicitly attend to values of health promotion when seeking to improve dietary outcomes.

### Strengths and Limitations

Our study provides one of the first in-depth accounts of the values of a diverse group of health professionals in the Netherlands and is a contribution to making the values that inform efforts to improve healthy eating more explicit. As health professionals are often unaware of the values underlying practice, a strength of the study was the use of the RLCHPM as a conceptual framework. The RLCHPM offered a strategic tool to help us explore highly subjective concepts using a set of well-defined critical health promotion values. For example, having professionals consider where efforts to improve healthy eating should be focused exposed ethical values related to prioritizing action with the most disadvantaged populations. Similarly, values concerning ethical change processes were uncovered by having professionals reflect upon whether current strategies include the active participation of the populations affected by nutrition inequalities or if decisions were made externally. However, as some participants (*n* = 13) were recruited through personal networks of study team members, it is possible that social desirability bias influenced the results of the study and/or that participants shared similarities that made them more open to participating in an interview. It is also important to recognize that the results of the study are inevitably influenced by the interpretations of the researchers who completed the reflective TA. An inherent bias may be evident in our selection of the RLCHPM as a conceptual framework for the study, as this choice shows our support for action that is consistent with the current critical health promotion values and principles found in the model. Finally, as values cannot be observed directly, it is possible that the results of the study present an incomplete understanding of professionals’ health promotion values. As described earlier, we used strategies to increase trustworthiness in recognition of these limitations, including piloting the interview guide and sending a summary of the research findings to all participants.

## 5. Conclusions

By critically examining the values of a diverse group of health professionals involved in strategies to promote healthy eating in SDPs, this study has identified philosophical, ethical, and technical health promotion values that overlap and conflict. Holistic and conventional conceptualizations of health promotion co-exist in the Netherlands, translating into an enduring emphasis on SDPs being responsible for their own health despite the wide recognition that eating behaviours are influenced by a wide range of social and structural factors. As such, it is important to change direction from focusing on the eating behaviours of individuals and continue to critically reflect on the values of individuals and organizations that develop, implement, and evaluate healthy eating strategies. An understanding of individual and collective health promotion values—and the actions taken to enact these values in daily practice—can increase awareness of the assumptions underlying practice and reorient strategies to improve healthy eating in SDPs towards a more critical, ethical, and equitable approach.

## Figures and Tables

**Table 1:** Characteristics of research participants.

Characteristic	*n* ^1^
Self-identified gender	
Female	24
Male	5
Experience in current role (years)	
≤5	11
6–10	4
11–15	4
16–20	2
≥20	8
Profession	
General practitioner (GP)	7
Dietitian (D)	4
Academic (A)	6
Professional at health promoting institution (HP)	7
Professional in municipal public health services (MHS)	9

^1^ Four participants worked in more than one profession category; as such, the sum exceeds the total number of 29.

## Data Availability

The electronic data supporting results presented in this study are retained for a period of ten years at Wageningen University & Research. Original recordings and transcripts are not publicly available due to privacy restrictions.
